# Future-Proofing the Oral Health Workforce: Evidence From Four Low- and Middle-Income Countries on Training Needs in Dental Public Health

**DOI:** 10.7759/cureus.96276

**Published:** 2025-11-07

**Authors:** Abhishek Mehta, Manu Mathur, Richard Watt, Carol C Guarnizo-Herreño, Regina J Mutave, Paulo S Goes, Barsha P Deka, Shehnaz Khan, Michelle Stennett

**Affiliations:** 1 Public Health Dentistry, Faculty of Dentistry, Jamia Millia Islamia, New Delhi, IND; 2 Health Policy, Public Health Foundation of India, Gurugram, IND; 3 Epidemiology and Public Health, University College London, London, GBR; 4 Collective Health, Universidad Nacional de Colombia, Bogotá, COL; 5 Community and Preventive Dentistry, University of Nairobi, Nairobi, KEN; 6 Clinical and Preventive Dentistry, Universidade Federal de Pernambuco, Recife, BRA

**Keywords:** capacity building, competency-based education, health workforce, low- and middle-income countries, oral health

## Abstract

Background: The dental public health workforce in low- and middle-income countries (LMICs) faces challenges including limited resources, a shortage of personnel, uneven distribution, and inadequate training opportunities. Obsolete educational frameworks and regulatory barriers exacerbate the problem further. A training needs assessment (TNA) is a crucial exercise to identify gaps in the current training landscape before instituting any capacity-building initiatives.

Objectives: This study aims to identify existing postgraduate and short-course training opportunities in dental public health, assess gaps and barriers, and determine priority competency areas among four LMICs (India, Colombia, Brazil, and Kenya).

Materials and methods: Following consultations with relevant stakeholders, a structured, theme-based tool for TNA was developed. The data were collected in each country through online and offline searches as deemed appropriate. Data from each theme were analyzed, and a comparative analysis by country was prepared.

Results: Across participant countries, postgraduate dental public health training opportunities varied considerably, with differences in program availability and accessibility. The availability of short courses was inconsistent, highlighting gaps in professional development opportunities. Key training priorities identified were qualitative research methods, community engagement, health advocacy and policy, health economics, academic publishing, grant proposal preparation, health promotion, and epidemiology. Barriers to further training included costs, limited postgraduate opportunities, and regional disparities.

Conclusions: This research highlighted significant gaps in dental public health workforce training across four LMICs, underscoring the need for a more equitable distribution of training resources, competency-based education, and an innovative workforce model that fosters strong interdisciplinary collaboration. Strengthening south-south collaboration, improving working opportunities and conditions, and expanding awareness of non-clinical career pathways are essential for building a resilient and impactful workforce in these countries.

## Introduction

The growing crisis of oral diseases has shifted global health priorities, transforming oral health from being a low priority on the global and national health agendas into an urgent issue that demands strategic action and systemic reforms. Over the last three decades, the prevalence of preventable oral diseases has surged by 50% [1], showing no signs of abating. The consequences of inadequate oral health policies disproportionately affect the global south, particularly the low- and middle-income countries (LMICs), where conditions such as dental caries, periodontal diseases, and oral cancers are widespread and increasing [1,2]. These LMICs face a critical shortage of trained oral health professionals who are equipped to design, analyze, and implement effective public health interventions. Even in contexts where workforce numbers are adequate, limited work opportunities and restrictive public policies constrain their ability to address the substantial burden of oral diseases and associated inequalities [2]. A dominant focus on clinical dentistry, insufficient funding, and limited career opportunities in research, as well as inadequate training in contemporary dental public health methodologies, hinder the quality of oral health research emerging from these regions. Institutional factors, including extended timelines for certain ethical and administrative approvals, evolving regulatory frameworks, resource constraints, and an urgent need for curriculum reforms, present additional challenges [3,4]. These trends underscore the importance of capacity-building initiatives that enhance training and workforce development within national health systems and global oral health initiatives.

A landmark effort to assess global oral health workforce capacity was led by the Oral Health Working Group (OHWG) of the World Federation of Public Health Associations (WFPHA). Acknowledging the limited availability of workforce data, OHWG conducted a global survey to map public health education and the integration of the oral health workforce into national health systems, revealing critical gaps in infrastructure and reinforcing the need for a structured, competency-based approach to workforce development [5]. The WHO Global Oral Health Action Plan (GOHAP) also highlights that achieving universal health coverage (UHC) depends on a well-trained and equitably distributed health workforce [6]. Frenk et al.'s influential work on transforming health professional education stressed the importance of developing globally competent yet locally relevant professionals with adaptable skills to meet regional and global health needs. While this concept has gained wide acceptance, there remains a pressing need to contextualize competency-based training for LMICs, where structural and resource limitations pose significant challenges [7,8]. To develop such a competent workforce, a structured reform of the existing curriculum and other system reforms are required.

This exploratory review was performed as a preliminary step before designing and developing a training and capacity-building program under the "Project CORE (Community Focused Oral Health Research for Equity)," a multi-country initiative aimed at reducing oral health disparities, addressing commercial determinants of oral health, strengthening oral health systems, and enhancing the research and programmatic capacity of the next group of oral health researchers in LMICs in Colombia, Brazil, India, and Kenya [9]. Before initiating these capacity-building activities, we conducted a structured training needs assessment (TNA) to identify priority training areas within dental public health at the national level in these countries.

## Materials and methods

Study design

This study is a multi-country exploratory TNA as part of Project CORE, an NIHR-funded research program in the United Kingdom. It was designed to map postgraduate and continuing education opportunities in dental public health, identify gaps in training, and document barriers that hinder both the development and acquisition of high-quality modern training in the field across participating countries. The study is descriptive in nature, conducted to conduct a baseline situational analysis. It combines structured desk research with stakeholder input to ensure contextual relevance.

Study population and scope

The study did not involve individual human participants; instead, the “population” under review consisted of postgraduate programs, short courses, and professional training opportunities related to dental public health. National stakeholders and global oral health experts were consulted during the tool development process to validate the themes and ensure contextual relevance.

Development of the TNA tool

A structured, theme-based TNA tool was developed through a multi-pronged process. First, a review of prior TNAs in health professions was conducted to identify core domains. An initial draft questionnaire was prepared and circulated among international dental public health experts and national stakeholders for feedback. Based on their inputs, revisions were incorporated through an iterative process. The final tool included four main themes: Postgraduate programmes in dental public health, which focused on mapping existing formal education opportunities; Availability of short courses, aimed at identifying continuing education and professional development options; Training needs priorities, which documented underrepresented or missing topics of high importance; and Barriers to training, which captured structural, economic, and contextual challenges to capacity-building (see Table in Appendices).

Data collection

Data were collected between November 2024 and January 2025 through a systematic online and offline search of institutional websites, regulatory bodies, training listings, grey literature, and freely available international resources, including PAHO/WHO courses. National investigators conducted searches in English and local languages (Spanish in Colombia, Portuguese in Brazil, and English in India and Kenya), focusing on postgraduate programs, short courses, training priorities, and barriers to dental public health education. The data gathered included the type and duration of training, number of seats, fee structures, and geographic distribution, providing a comprehensive overview of the existing training landscape and its gaps.

We included accredited postgraduate and short courses in dental public health, excluding unverified, informal, or non-relevant programs. As this study primarily relied on publicly available data and did not involve direct interaction with human participants, formal institutional ethics approval was not required.

Analysis and presentation of data

Information on postgraduate courses and training opportunities was systematically compiled in Excel (Microsoft Corp., Redmond, WA, USA) and categorized by country. Training offerings were mapped to identify the scope and distribution of available programs across regions. Each country and key thematic areas then organized the results to facilitate comparative assessment. Priorities were identified for each country from both a global and local context. This approach helped highlight both common and country-specific gaps, which will inform the development of targeted training strategies.

## Results

Thematic analysis was conducted to identify broader themes and sub-themes, which are summarized in Table 2. Detailed results of each theme are discussed as follows:

**Table 1:** Thematic analysis of dental public health TNA exercise in four LMICs (India, Kenya, Brazil and Columbia) This table is the authors' own creation and is not reproduced from any published source. LMICs: low- and middle-income countries, PAHO: Pan American Health Organization, WHO: World Health Organization, TNA: training needs assessment

S. no.	Themes	Sub-themes	Findings
1.	Postgraduate program in dental public health	a. Availability and accessibility	Wide variation across countries: India offers postgraduation in public health dentistry, but mostly in private institutions. Colombia offers general public health MSc/PhD programs, but does not have specific courses in dental public health. Kenya offers a diploma in community oral health, but does not have a master’s program. Brazil has 7,028 postgraduate programmes overall, but only 3 in dental public health.
		b. Regional disparities	Brazil: concentration of programme in the Southeast region; Colombia and Kenya: limited access in rural areas.
		c. Duration	All countries: course duration varies widely, from 1-year diplomas to 5-year PhDs. Colombia demonstrates greater flexibility, offering both technical training programs and specialized courses, which are supported by PAHO/WHO.
2.	Short courses availability	a. Subject areas	India: stronger in biostatistics and research methodology, but gaps exist in qualitative methods and health economics. Colombia: courses in epidemiology, admin management, and public health nutrition. Kenya: strong in research methods, monitoring and evaluation, leadership/governance. Brazil: strong in epidemiology, health policy, human and social sciences, mostly at public universities.
		b. Accessibility and cost	Education is largely provided by public institutions, particularly in Brazil, with tuition often free. However, there are persistent gaps in interdisciplinary skill development and training.
3.	Training needs priorities	a. Research competencies	All countries prioritized qualitative and quantitative research methods, epidemiology, data analysis, and academic publishing.
		b. Policy and advocacy	Health advocacy and policy are identified as critical, particularly in India, Colombia, and Kenya.
		c. Community engagement	Lack of structured training in all four countries.
		d. Other priorities	Health promotion, grant writing, and health economics were emphasized variably across the contexts.
4.	Barriers to capacity building	a. Economic barriers	High tuition costs remain a challenge in the private sector across India, Colombia, Kenya, and Brazil. In Brazil’s underserved regions, the expense of devices and internet access is a barrier.
		b. Structural/institutional	India and Kenya: insufficient opportunities for hands-on and practical training. Across all countries: limited availability of mentorship and supervision support.
		c. Geographic inequities	Training concentrated in urban centers, particularly in Colombia, Kenya, and Brazil.
		d. Opportunities	Kenya: professionals are often required to leave employment and pursue postgraduate education abroad due to limited local options. Colombia: postgraduate programme offers limited flexibility for working professionals.
		e. Infrastructure barriers	Brazil: unstable electricity and internet access in rural regions.

Available postgraduate programs related to dental public health

**Figure 1:**
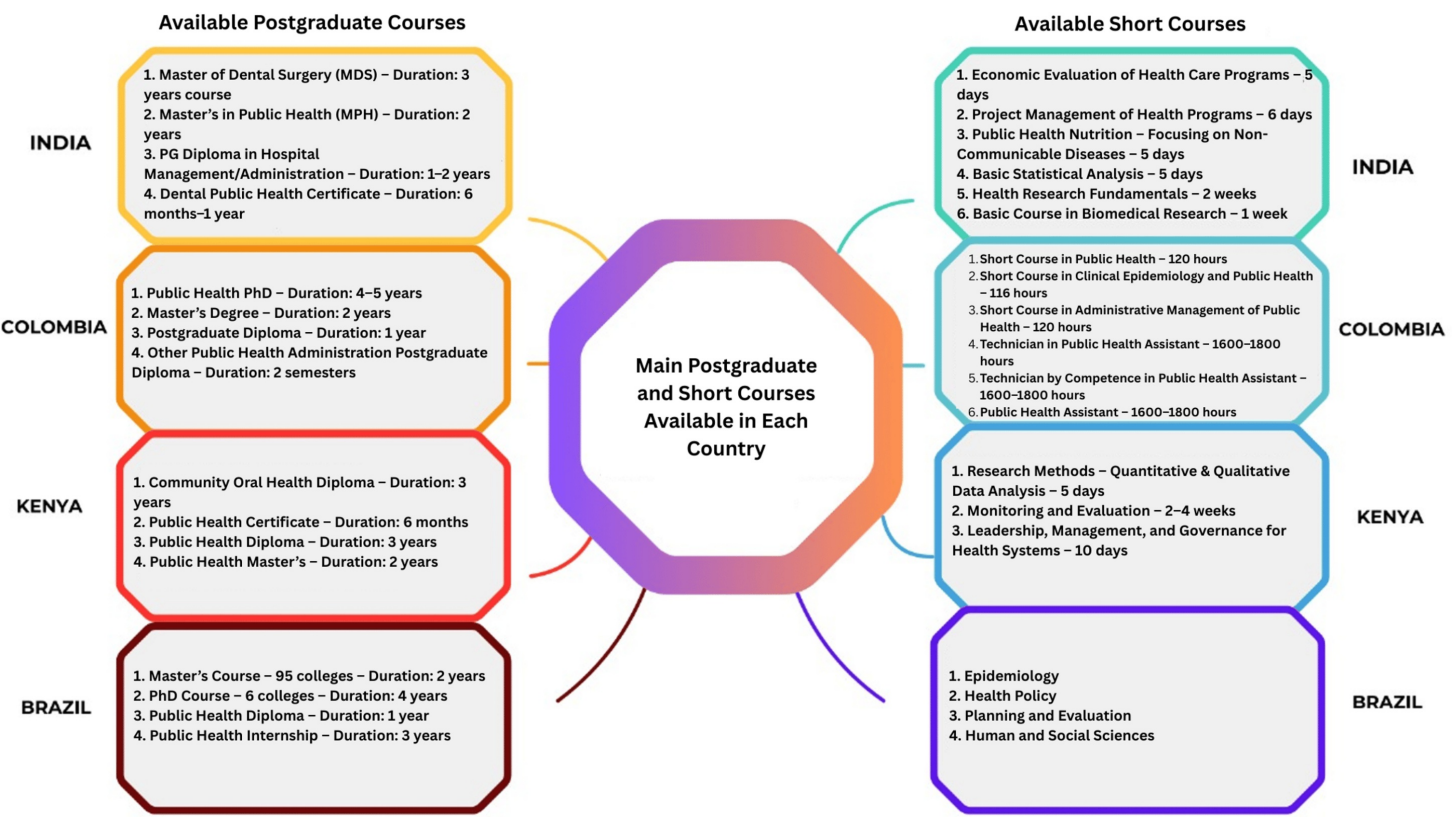
Main postgraduate and short courses available in each country This figure is the authors' own creation and is not reproduced from any published source.

Postgraduate education and training opportunities varied significantly across each participating country, reflecting differences in accessibility, program availability, and specialization (Figure 1). In India, the primary postgraduate program in dental public health is the master of dental surgery, a three-year course with an annual intake of 267 seats (31 government and 236 private) [10]. In Colombia, a broader range of public health and technical education programs is available, including PhDs (four to five years), master’s degrees (two years), and postgraduate diplomas (one year). While no specific dental public health program exists, Colombia has a well-established tradition of public health training, with 22 master’s and 4 PhD programs as of 2023. Technical training under the education for work and human development framework offers flexible, practical education across 19 private institutions. Additionally, free international courses are available online through PAHO/WHO [11-13]. Kenya lacks a dedicated master’s program in dental public health, and training is instead offered through a three-year diploma in community oral health in two institutions. Public health qualifications range from short-course certificates (six months to one year) offered at seven institutions, master’s degrees (two years) available at six universities, and PhD programs offered at four universities. Brazil’s postgraduate education system is the most expansive, with 7,028 programs, including 205 master’s and 116 doctoral courses. However, regional disparities persist, with the Southeast region hosting 94 programs compared to only 12 in the North. Dental public health training remains scarce, with only three programs, all of which are concentrated in the southeast. Public health education, by contrast, is more widespread, with 100 programs available. Brazil categorizes collective health into three domains: epidemiology, health policy, and human and social sciences, with epidemiology being the most represented [14-16]. Collectively, these findings highlight considerable variation in postgraduate education across these countries, particularly in the availability and accessibility of dental public health training.

Short courses availability

The findings revealed notable disparities across subject areas in short courses. Some of the prominent short courses available in each country are mentioned in Figure 1. Courses in India, such as research methodology and biostatistics, appeared to be well-established and more commonly offered. In contrast, short courses in areas such as qualitative research methods and health economics were less prevalent, indicating gaps in access to key interdisciplinary skills essential for public health and research [17,18]. In Colombia, varying levels of short courses were identified, with the most recognized programs including public health, clinical epidemiology, and administrative management of public health. Advanced training options for public health assistants, such as a technician in public health assistant and public health assistant programs, were also highlighted. Kenya’s short course availability revealed a significant interest in research methods, quantitative and qualitative data analysis, monitoring and evaluation, as well as leadership, management, and governance for health systems. In contrast, in Brazil, short courses in epidemiology, health policy, planning and evaluation, and human and social sciences were most common, with epidemiology being the most prominent. Most public health and dental public health courses are offered by public institutions, where education is provided free of charge, with 88 programs operating under this model [15]. These findings underscore the diverse levels of awareness and access to specialized training across the four countries.

Training needs priorities

**Figure 2:**
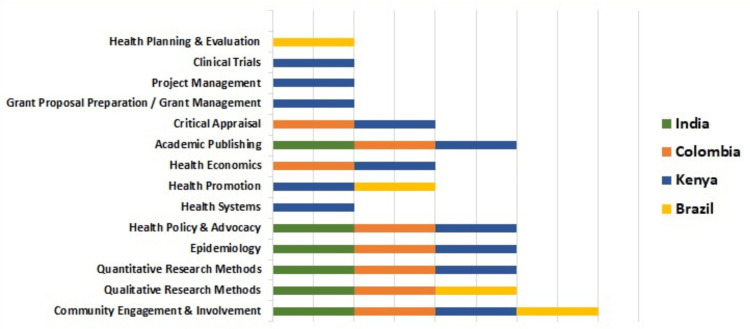
List of common and exclusive competencies prioritized across each country This figure is the authors' own creation and is not reproduced from any published source.

Based on the conducted search, a list of training needs priorities was compiled for each country. Formal training in community engagement and involvement was lacking in all four study countries. In contrast, research methodology courses (both qualitative and quantitative), epidemiology, health policy and advocacy, and academic writing training were priorities in at least three countries. Kenya had the highest priority for courses related to dental public health (Figure 2). These findings highlight the diverse and evolving training needs essential for strengthening public health capacities across countries.

Barriers to capacity building

**Figure 3:**
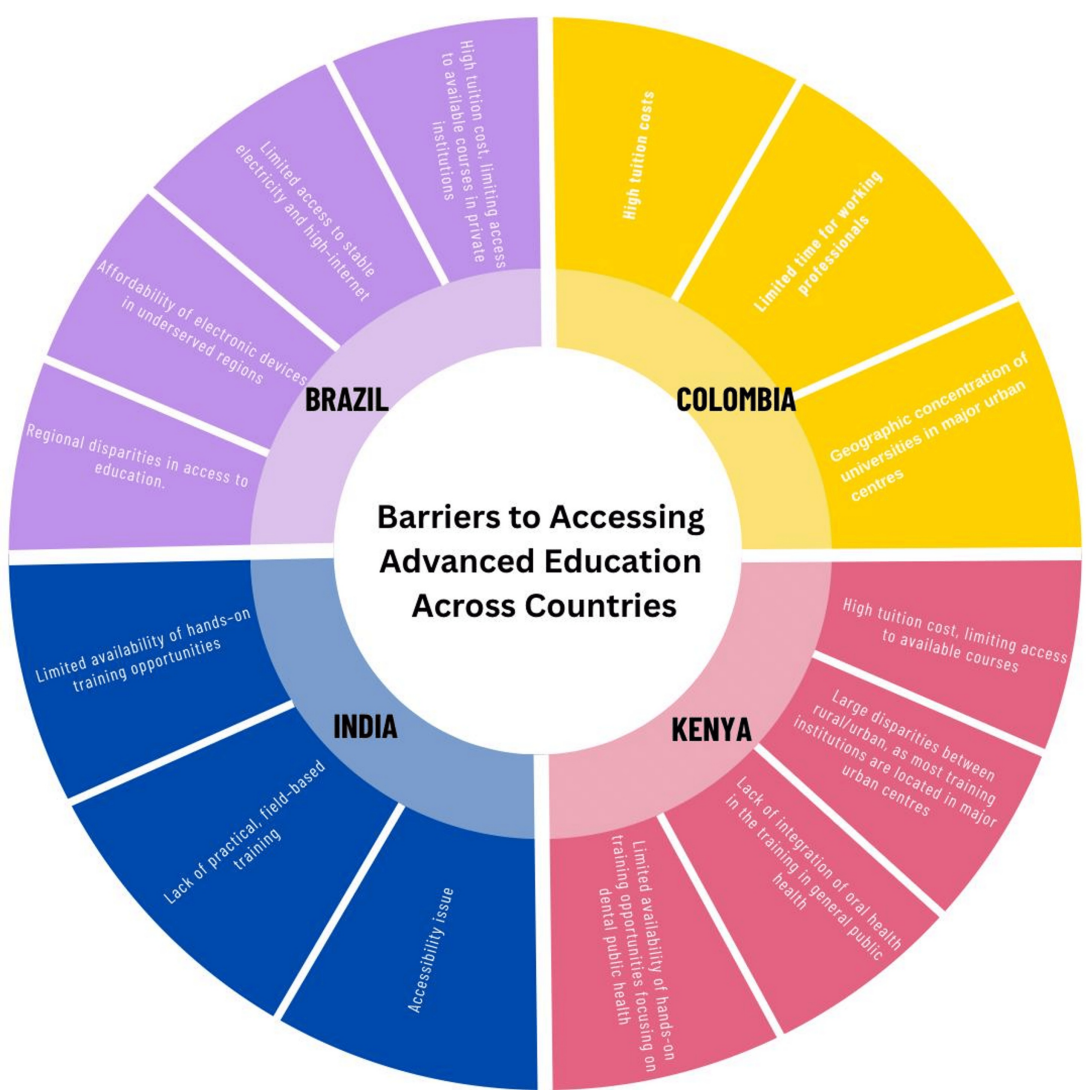
Barriers to accessing advanced dental public health training across countries This figure is the authors' own creation and is not reproduced from any published source.

Training barriers varied across the participating countries and were shaped by structural, economic, and regional disparities. Common barriers across all four participating countries were high course fees, limited access to training courses, and regional disparities (Figure 3). Other country-specific barriers, for example, in India, include a lack of hands-on training, which has been identified as a limitation to developing practical, field-based skills. Additional challenges included the unequal distribution of postgraduate seats between public and private dental institutions, along with significant differences in fee structures, which affect access for students from lower socioeconomic backgrounds and remote regions [10,19-20]. In Colombia, there was limited flexibility for working professionals to upgrade their knowledge. Blended mentorship models were identified as a potential solution, particularly for areas that require in-person training, such as qualitative research methods and community engagement. In Kenya, postgraduate dental public health programs are unavailable locally, often requiring practitioners to leave clinical work and relocate, typically to South African universities for extended periods, making such programs financially and logistically inaccessible [21]. In Brazil, despite the availability of free public education, regional inequalities persist in restricting access. While hybrid and distance-learning models offer potential, underserved regions still face significant barriers, including the high cost of electronic devices, inconsistent electricity supply, and limited access to high-speed internet. These findings underscore the need for more inclusive, accessible, and flexible training models tailored to diverse local contexts.

## Discussion

This research provides a comprehensive examination of the competency gaps in the dental public health workforce across four emerging economies worldwide, employing a multi-pronged approach to TNA. Despite distinct national contexts, common themes emerged, including disparities in educational access, gaps in interdisciplinary training, and barriers to participation in specialized programs. The third strategic objective of the World Health Organization’s GOHAP 2023-2030 [5] highlights the need to develop innovative workforce models and expand competency-based education to address population oral health needs. By identifying priority competencies and key barriers, this study provides evidence-based recommendations to enhance oral health workforce development by addressing the training needs that have been identified.

Lomazzi et al. highlighted the lack of capacity-building and training programs for the oral health workforce globally [3]. Nearly a decade later, our findings indicate that this gap persists. Within the scope of oral health, LMICs have traditionally prioritized individual clinical care over population-based preventive and promotive care, as well as action on the social, political, and commercial determinants of oral health. This has led to a lack of resources and investment in training programs that equip the oral health workforce, particularly in dental public health, with the essential skills needed to address the rising burden of oral diseases and related inequalities [3,5-6]. In India, the government-funded postgraduate programs in dental public health are severely limited, with private institutions offering more than five times as many seats.10 This disparity limits equitable access, especially for students from lower socioeconomic backgrounds. Beyond program availability, significant financial barriers remain, including high tuition fees in Colombia and the costs of electronic equipment and internet access in Brazil [22].

Although a large number of short courses are now available through online platforms, offline and hybrid training formats remain the preferred options across countries. In India, there is a clear preference for in-person training, likely due to the perceived effectiveness of hands-on learning and face-to-face mentorship. In Brazil and Colombia, hybrid models are favored to address accessibility barriers, as most training programs are concentrated in major urban centers, particularly in the southeastern region of Brazil and the metropolitan areas of Colombia. In Kenya, the introduction of in-country dental public health courses could enable wider participation through hybrid formats, allowing professionals to balance clinical duties with flexible learning schedules that include virtual classes and periodic in-person sessions for collaborative engagement. These preferences highlight the importance of flexible, context-sensitive delivery methods that go beyond the convenience of online access alone.

Concerning competency priorities, training in qualitative research methods and epidemiology emerged as a common priority across India, Kenya, and Colombia, reflecting a key gap in the current training. Limited oral health data in LMICs has long hindered evidence-based decision-making and policy integration [23]. Strengthening epidemiological skills can help bridge this gap by enabling the workforce to generate essential data, monitor disease trends, and advocate for better oral health policies. Additionally, training in quantitative analysis and critical appraisal skills was consistently prioritized across all countries, while training for academic publication emerged as a key priority in India, Kenya, and Colombia. The analysis conducted in India and Kenya identified a lack of methodological expertise as a key barrier to academic publication [21-20,24]. The absence of a robust research culture across educational institutions, compounded by administrative limitations and inadequate faculty support due to competing teaching commitments, has been widely recognized as a major barrier to research development across all the study countries. Furthermore, our study identified community engagement and involvement as a key priority, reinforcing the growing emphasis on community-focused and implementation research in LMICs.

Given the paucity of foundational research competencies outlined above, there is a need to move beyond traditional knowledge-based training and build locally appropriate alternatives that combine the structure of a competency-based approach with participatory, problem-posing methods inspired by critical pedagogy [25]. This shift would equip the oral health workforce with practical, system-oriented skills that align with real-world health needs and evolving public health challenges. The GOHAP recommends developing a standardized, national competency-based training curriculum for oral health professionals, aligned with the WHO’s Global Competency and Outcomes Framework for UHC [5,24].

Addressing the shortage of trained professionals requires a multi-faceted approach that incorporates interdisciplinary team-based learning and interprofessional collaboration [5-7]. Strengthening collaboration across disciplines can facilitate knowledge sharing and joint capacity-building initiatives, particularly through south-to-south partnerships. Peer-led learning networks can further compensate for gaps in mentorship and expertise while fostering sustainable workforce development. The GOHAP also highlights the importance of reforming intra- and inter-professional oral health education to keep training models responsive to evolving health needs. Furthermore, ensuring that workforce competencies align with public health priorities, such as integrating oral health into primary care settings, will enhance service delivery and long-term sustainability [5].

While this research provides valuable insights into the training needs of the dental public health workforce in LMICs, certain limitations must be acknowledged. Certain variations in data collection methods across countries, stakeholders' representation, and reliance on secondary data present a limitation in terms of standardization. However, this methodological diversity also serves as a strength, as it allows the study to accommodate the unique logistical constraints of each country, ensuring broader participation. Notably, a key strength of this study lies in its evaluation of existing programs, thereby addressing a critical gap in the literature on dental public health workforce development.

## Conclusions

This research underscores the persistent gaps in dental public health workforce training across LMICs, highlighting the need for competency-based education, interdisciplinary collaboration, and innovative workforce models. Barriers, including financial constraints, limited mentorship, and unequal access to training opportunities, continue to impede workforce development. Strengthening south-south collaboration is crucial for promoting cooperation and knowledge sharing. Additionally, raising awareness of non-clinical career pathways, such as public health, epidemiology, and health policy, is equally important in strengthening the workforce and broadening its impact. Future research could focus on identifying specific skills within the broader competency areas and evaluating their relative priority. This would help inform the development of a structured training program framework tailored to the needs of the oral health workforce in different contexts.

## Appendices

**Table 2:** Training and capacity building needs assessment data collection tool

Section category	Course type/category	Provider	Duration	Approx. cost	Modality (in-person/online/blended)	Language	Barriers to access	Priority ranking (from 1-10)	Reason for priority
A.1 (Postgraduate courses in dental public health/public health)	Stand-alone modules								
	Certificate								
	Diploma								
	Masters								
A.2	Short courses/CPD								
A.3	Training opportunities/capacity building initiatives								
A.4 (Priority training needs identified)	Community engagement/development								
	Qualitative research methods								
	Quantitative research methods								
	Statistical analysis								
	Critical appraisal								
	Health policy and advocacy								
	Academic publishing/grant writing								
	Epidemiology								
	Health systems								
	Health promotion								
	Health economics								
